# Preschool Teachers’ Intentions to Use GenAI: Extending UTAUT

**DOI:** 10.3390/bs16060840

**Published:** 2026-05-22

**Authors:** Chenchen Hao, Zeguo Wang, Ping Wang

**Affiliations:** 1 Faculty of Education, Northeast Normal University, Changchun 130024, China; wangp@nenu.edu.cn; 2 School of Information Science and Technology, Northeast Normal University, Changchun 130024, China; wangzg838@nenu.edu.cn

**Keywords:** generative artificial intelligence, preschool teachers, UTAUT model, behavioral intentions

## Abstract

Against the backdrop of the rapid development of generative artificial intelligence (GenAI), exploring preschool teachers’ willingness to adopt technology is critical for advancing their educational applications. However, this area remains underexplored. To address this gap, this study draws on the Unified Theory of Acceptance and Use of Technology (UTAUT) to develop a research model incorporating performance expectancy, effort expectancy, social influence, facilitating conditions, perceived risks, and tech-savviness. Using a sequential mixed-methods design, we recruited 434 teachers to participate in a GenAI teaching-application workshop, collected 399 valid questionnaires for structural equation modeling, and conducted 15 in-depth interviews. Quantitative results indicate that performance expectancy, social influence, and tech-savviness are positively associated with preschool teachers’ intention to use GenAI, while perceived risk is negatively associated; effort expectancy and facilitating conditions show no significant association. Due to methodological limitations including high inter-construct correlations and potential common method bias, these findings should be viewed as exploratory rather than conclusive. Qualitative interviews support these relationships and provide further explanatory insights. The mixed-methods results offer preliminary hypotheses regarding GenAI adoption among preschool teachers, and future confirmatory research is needed to verify their generalizability, especially in collectivist cultural contexts.

## 1. Introduction

The rapid advancement of generative artificial intelligence (GenAI), particularly large language models, has garnered significant interdisciplinary attention (Hyder et al., 2019). In education, GenAI is increasingly recognized for its transformative potential, offering capabilities such as personalised learning experiences (Elbanna & Armstrong, 2024; M. Liu et al., 2023; Rahiman & Kodikal, 2024; Su & Yang, 2022), real-time feedback and assessment (Hwang & Chang, 2023; Elbanna & Armstrong, 2024), and support for data-driven instructional decision-making (Boscardin et al., 2024). These applications collectively suggest that GenAI could become an indispensable tool for educators, students, and researchers alike. At the same time, it demonstrates substantial potential in helping non-native learners overcome language barriers (Alasadi & Baiz, 2023). However, the educational value of information technology can only be realized when users actively adopt and apply it (Venkatesh & Davis, 1996). Existing studies indicate that teachers’ willingness to use and their acceptance of educational technologies play a crucial role in their successful implementation (Yuen & Ma, 2008).

Yet, prior research has primarily focused on pre-service preschool teachers’ perceptions and attitudes toward artificial intelligence (Lamanauskas, 2025; Korkmaz & Sagnak, 2024), while studies examining how in-service preschool teachers perceive and utilize GenAI remain notably scarce. Research consistently identifies teachers as the most influential factor shaping early childhood education quality and children’s developmental outcomes (Fagot, 1973; Hamre et al., 2014; O’Connor & McCartney, 2007). GenAI amplifies this influence. Teachers must interpret AI-generated content, adapt it for young learners, and protect children from risks like inaccurate information or data privacy breaches (Chen & Lin, 2024; Kurniah et al., 2023). GenAI offers multi-level benefits—improving lesson planning efficiency, generating content, and supporting personalized learning (Chatterjee & Bhattacharjee, 2020; Elbanna & Armstrong, 2024). However, early childhood education presents unique challenges. Unlike elementary or secondary education’s content-driven, standardized curricula, preschool emphasizes play-based learning, social-emotional development, and holistic growth (Su & Yang, 2022). Therefore, educators must carefully adapt GenAI tools and outputs to ensure developmental appropriateness and protect young children’s well-being (Chen & Lin, 2024). For these reasons, we cannot simply apply findings from other grade levels to preschool teachers, highlighting the need for targeted research on this population. Yet China’s Professional Standards for Preschool Teachers (Ministry of Education, 2012) does not specify requirements for information technology competencies, revealing a systematic policy gap in supporting teachers for this critical role. Therefore, systematically investigating the key factors that influence preschool teachers’ use of GenAI holds significant theoretical value and practical urgency for promoting its effective and appropriate application in early childhood education.

Regarding methodology, most existing studies have adopted the Technology Acceptance Model (TAM), focusing on perceived usefulness, ease of use, and attitude toward usage behavior (Kong et al., 2024; G. Liu & Ma, 2024; Yilmaz et al., 2023; Sallam et al., 2023). Although TAM has been widely validated in educational settings (Pynoo et al., 2011; Teo & Noyes, 2014), it insufficiently considers social and institutional factors (Gao & Gao, 2011). This limitation is especially notable in early childhood education, where teachers’ decisions are affected by peer norms, administrative expectancies, and institutional support (Hong et al., 2021). Building on this, Venkatesh et al. (2003) proposed the Unified Theory of Acceptance and Use of Technology (UTAUT), which has demonstrated strong explanatory power for technology adoption in higher education (Xue et al., 2024). Nevertheless, its application in the K-12 education context remains underexplored (Xue et al., 2025). In particular, within the context of GenAI, UTAUT requires appropriate adaptation or contextualization to account for the specific characteristics of early childhood education.

Against this background, this study focuses on preschool teachers as a critical yet understudied group and employs the UTAUT model as its analytical framework. To explore the model’s potential within the GenAI and early childhood education context, we extend UTAUT by incorporating two additional constructs—perceived risks and tech-savviness. Perceived risks capture teachers’ concerns regarding data security and content reliability, which are particularly salient when working with young children (Chen & Lin, 2024). Tech-savviness reflects teachers’ operational competencies and confidence with technology, which may shape their capacity to effectively leverage GenAI tools (Demir & Demir, 2023; M. Zhang et al., 2022). By integrating these constructs, this study offers a preliminary description of factors that may be associated with preschool teachers’ intentions to adopt GenAI for learning and teaching. The findings offer preliminary insights into preschool teachers’ technology acceptance mechanisms and suggest possible directions for strategies to promote GenAI use in early childhood education. Given the cross-sectional and single-source nature of the data, this study adopts an exploratory rather than confirmatory approach. We do not claim causal inference or distinct construct separation, but aim to inform future research on preschool teachers’ GenAI adoption with a cautiously interpreted empirical model.

## 2. Literature Review

### 2.1. GenAI in Early Childhood Education

GenAI and its implications for children’s development have been subjects of sustained scholarly discussion (Doroudi, 2023). However, with the rapid advancement of GenAI, its applications in early childhood education have begun to attract renewed and widespread academic attention. The capabilities of GenAI in generating text, videos, and images open new possibilities for creating personalized and creative learning environments for children (Shazaad & Harrison, 2025), thereby catalyzing a growing body of related research.

GenAI technologies have great potential to support early childhood education (Su & Yang, 2022; Su & Zhong, 2022). GenAI can create culturally relevant content and personalize learning resources (Baskara, 2023). GenAI conversational agents such as ChatGPT offer tailored support through interactive dialogue (Chen & Lin, 2024). Educational robots and chatbots can promote children’s inquiry, creativity, emotional skills, and collaboration (Lin et al., 2020; Kewalramani et al., 2021; Vartiainen et al., 2020; Williams et al., 2019). However, these benefits also raise legitimate developmental concerns. Overuse of AI may hinder children’s social development and problem-solving skills without proper guidance (Chen & Lin, 2024). Issues such as inaccuracies and biases in AI-generated content remain unsolved (G. L. Liu et al., 2024). This tension between opportunities and risks highlights the essential role of teachers. Preschool teachers interpret, contextualize, and monitor AI content for young children. They can unlock GenAI’s pedagogical value while reducing developmental risks. Their informed, context-aware decisions about integrating GenAI will determine whether the technology benefits early childhood education or causes unintended harm.

Existing studies on preschool teachers’ technology acceptance have largely focused on the COVID-19 pandemic period. For example, Hong et al. (2021) and Velki and Miočić (2023) respectively examined teachers’ willingness to adopt educational technologies during this exceptional period and the factors influencing their acceptance. N. A. A. Ahmad and Zabadi (2022) employed the TAM to examine the technology acceptance of Palestinian preschool teachers during the COVID-19 pandemic. However, these studies generally adopted the TAM framework and focused on conventional educational technologies rather than GenAI. Within the context of early childhood education, systematic research on how in-service teachers understand, accept, and apply GenAI remains scarce. This gap provides a clear point of departure for the present study.

### 2.2. Unified Theory of Acceptance and Use of Technology (UTAUT)

In 2003, Venkatesh et al. (2003) proposed the Unified Theory of Acceptance and Use of Technology (UTAUT), which integrates the strengths of eight models: the Theory of Reasoned Action (TRA), the Theory of Planned Behavior (TPB), the Technology Acceptance Model (TAM), the Innovation Diffusion Theory (IDT), the Motivational Model (MM), the Model of PC Utilization (MPCU), the Task-Technology Fit (TTF) model, and the Social Cognitive Theory (SCT). UTAUT demonstrates high accuracy in predicting users’ behavioral intention and actual technology use, with its predictors accounting for up to 70% of the variance (Oye et al., 2014), making it one of the mainstream theoretical models for examining factors influencing teachers’ technology adoption (Kemp et al., 2019).

**Figure 1:**
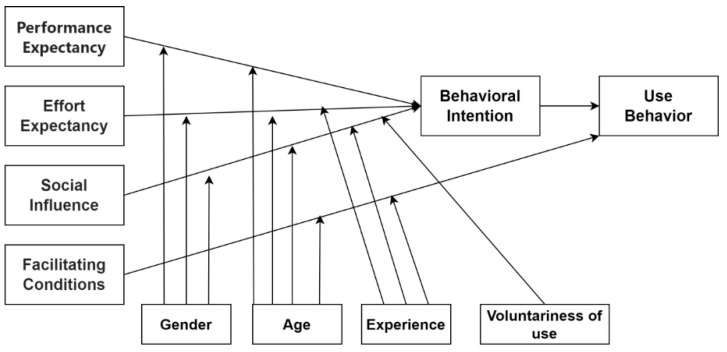
UTAUT key constructs (Venkatesh et al., 2003, p. 447).

The UTAUT model condenses the 32 variables from the eight models into a streamlined framework consisting of four main effects and four moderating factors. The four main effects are performance expectancy, effort expectancy, social influence, and facilitating conditions, while the four moderating factors—gender, age, experience, and voluntariness—are incorporated into the model and hypothesized to moderate the effects of the four constructs (performance expectancy, effort expectancy, social influence, and facilitating conditions) on behavioral intention and actual use (see Figure 1).

A substantial body of empirical research has employed UTAUT to examine the determinants of technology adoption, typically by extending the original model with additional core variables or moderating factors (Venkatesh et al., 2016). For example, Conti et al. (2017) incorporated personality factors into the UTAUT framework and investigated, through a survey of 112 primary and secondary school teachers, their willingness to use robots for instructional purposes. Jadil et al. (2021) conducted a quantitative meta-analysis of 127 studies on mobile banking adoption and tested the moderating effects of sample size and cultural context. Blut et al. (2022) synthesized 1451 studies to validate and extend UTAUT, revealing the moderating roles of technology types and cultural dimensions. Theres and Strohmeier (2023) further adapted the model to specific research contexts by introducing user attitudes and by incorporating moderating variables such as organizational sector, respondents’ background, and regional context.

Within the field of education, UTAUT has been primarily used to investigate technology acceptance among learners across educational stages (Du & Lv, 2024; Yakubu et al., 2025; Tang et al., 2025), among primary and secondary school teachers (Watted, 2025; Li & Zhang, 2024), and among university faculty (Zaim et al., 2024). The model’s four core constructs—performance expectancy, effort expectancy, social influence, and facilitating conditions—have proven effective in assessing individuals’ behavioral intention to use GenAI tools. However, research employing UTAUT to examine preschool teachers as a distinct professional group remains notably limited. The present study seeks to contribute to expanding the model’s application scope by investigating preschool teachers’ behavioral intention to use GenAI tools.

Although UTAUT2 (Venkatesh et al., 2012) extends the original model by incorporating hedonic motivation, price value, and habit, we opted for the original UTAUT framework in this study. Our study focuses on core determinants of technology acceptance in educational contexts, where performance and social factors play a central role. We also extended the UTAUT model by adding perceived risks and tech-savviness, which are highly relevant to early childhood education regarding child safety and teachers’ digital competence. Including all UTAUT2 constructs would have oversaturated the model and shifted attention away from these context-specific extensions.

### 2.3. Research Hypotheses

**Figure 2:**
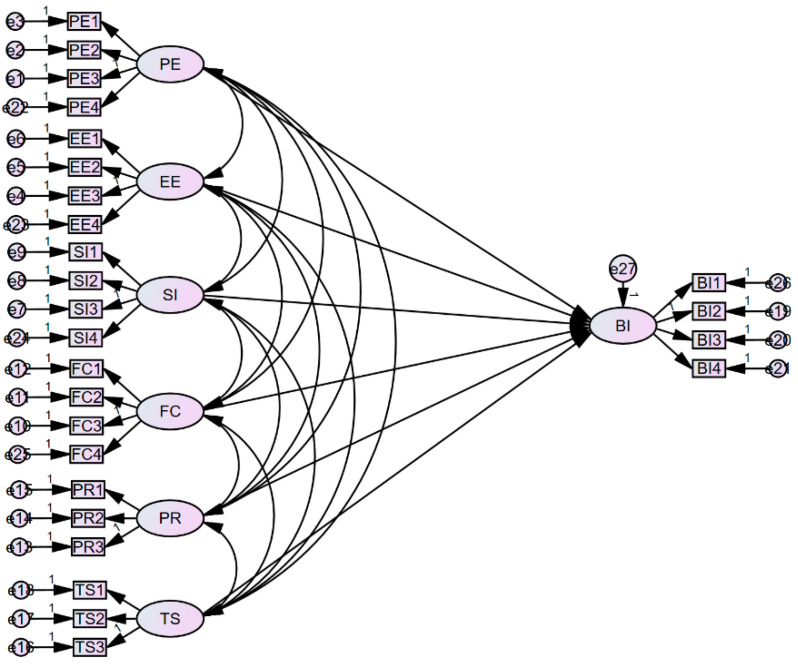
Research Model. **Note(s):** Performance expectancy (PE); Effort expectancy (EE); Social influence (SI); Facilitating conditions (FC); Perceived risks (PR); Tech-savviness (TS). All subsequent abbreviations in the text refer to the constructs listed above.

To enhance the explanatory power of the behavioral intention model for preschool teachers’ use of GenAI, this study extends the classical UTAUT framework by incorporating two additional constructs—perceived risks and tech-savviness—and develops an expanded analytical model (see Figure 2). This extension is justified both theoretically and contextually. Theoretically, UTAUT is a general technology acceptance model, but researchers have long recommended context-specific adaptations by including domain-relevant constructs (Venkatesh et al., 2016). In GenAI and early childhood education, two factors stand out: perceived risks (e.g., data security and content inaccuracy), which are critical given young children’s need for protection (Chen & Lin, 2024;), and tech-savviness, which captures digital competence and confidence in using GenAI effectively (Demir & Demir, 2023). Incorporating these variables yields a more comprehensive model explaining preschool teachers’ GenAI adoption intentions.

The present study does not include moderators, a decision supported both theoretically and empirically. Theoretically, this study focuses on the direct effects of core predictors on teachers’ behavioral intention, consistent with its exploratory aim in the understudied area of GenAI adoption in early childhood education. Empirically, including moderators is impractical: 98.5% of participants were female, providing insufficient gender variance. Additionally, the workshop design ensured uniform GenAI exposure, voluntary participation, and identical training, effectively controlling for experience and voluntariness. Thus, excluding moderators is a justifiable simplification that does not threaten the validity of the findings.

Performance expectancy refers to the belief that using a system improves job performance. Studies consistently show it positively predicts teachers’ technology adoption (Venkatesh et al., 2003; X. Zhang & Wareewanich, 2024; Pitpit et al., 2025). Effort expectancy captures how easy people perceive a technology to use (Venkatesh et al., 2003; Kohnke et al., 2023). Social influence describes the pressure individuals feel from colleagues or administrators to adopt technology (Venkatesh et al., 2003). Research confirms that peer and institutional support strongly predict teachers’ technology acceptance (Rwodzi & De Jager, 2021; Chiu, 2023). Facilitating conditions reflect the organizational and technical support people perceive as available for technology use (Venkatesh et al., 2003; Mohamed, 2024). Perceived risks involve concerns about negative outcomes like data breaches, inaccurate content, or privacy violations (Bauer, 1960). These concerns consistently reduce technology adoption across various contexts (Arfi et al., 2021; Wut et al., 2025; Widyanto et al., 2022). Tech-savviness describes individuals’ digital skills and confidence when using specific technologies (Apergis, 2019). It shapes how effectively people use technology (Demir & Demir, 2023; M. Zhang et al., 2022; Bankins et al., 2024). In this study, we define tech-savviness as combining practical digital skills with experience for confident technology use, aligning with modern digital competence frameworks. Alastor et al. (2025) conceptualised teacher digital competence as multi-dimensional, including technological literacy, information processing, and innovation. They argue that domain-specific self-perceived skills best capture this construct. Our measure, adapted from Guha et al. (2023), reflects this multi-faceted nature by assessing three aspects: ease using GenAI tools, being consulted for technical advice, and early technology adoption. These behavioral indicators distinguish tech-savviness from self-efficacy or general innovativeness.

Based on the aforementioned theoretical and empirical foundations, this study proposes the following hypotheses:

**H1.**  *Performance expectancy (PE) is positively associated with preschool teachers’ behavioral intention (BI) to use GenAI tools.*

**H2.**  *Effort expectancy (EE) is positively associated with preschool teachers’ behavioral intention (BI) to use GenAI tools.*

**H3.**  *Social influence (SI) is positively associated with preschool teachers’ behavioral intention (BI) to use GenAI tools.*

**H4.**  *Facilitating conditions (FC) are positively associated with preschool teachers’ behavioral intention (BI) to use GenAI tools.*

**H5.**  *Perceived risks (PR) are negatively associated with preschool teachers’ behavioral intention (BI) to use GenAI tools.*

**H6.**  *Tech-savviness (TS) is positively associated with preschool teachers’ behavioral intention (BI) to use GenAI tools.*

## 3. Methodology

### 3.1. Overview of the Research Process

To ensure data quality, this study adopted a sequential data collection procedure that integrated training, practice, and assessment. The process involved the following three stages.

Stage 1: Ethical approval and facilitator training

The research protocol was approved by the Northeast Normal University Faculty of Education Ethics Committee (No. NENU-FE-EC2025-073). The research team recruited and trained seven facilitators (four graduate students in educational technology and three in early childhood education). A two-day intensive workshop was conducted to standardise instructional content, operational procedures, and facilitation guidelines.

Stage 2: Teacher workshops

We recruited 434 preschool teachers to participate in a three-day in-person workshop on GenAI application in early childhood education. The workshop provided basic GenAI knowledge and hands-on practice. On Day 1, lectures covered core GenAI concepts, educational applications, and ethical issues. On Day 2, facilitators demonstrated three popular Chinese GenAI tools (DeepSeek, Doubao, and Tencent Yuanbao) and guided teachers through practical tasks including lesson planning, storytelling materials, parent communication, and classroom design. Teachers then completed hands-on tasks with step-by-step individual support. On Day 3, an open forum allowed teachers to ask questions, share experiences, and discuss challenges. To ensure consistency across sessions, seven facilitators completed a two-day standardized training covering scripts, protocols, response guidelines, and neutral facilitation, followed by a mock workshop and feedback.

Stage 3: Data collection

After the three-day workshop, we distributed an electronic questionnaire via Wenjuanxing to all participants, and data collection lasted four weeks. At the end of the questionnaire, we included an optional item inviting teachers to volunteer for a follow-up interview. Volunteers provided contact information on a separate anonymous form that was not linked to their questionnaire responses. In total, 87 teachers volunteered.

After the four-week data collection, we analyzed behavioral intention (BI) scores from 399 respondents and classified them into three groups: low (lowest 25%, scores ≤ 3.50), medium (middle 50%, 3.51–4.49), and high intention (highest 25%, scores ≥ 4.50). We purposively selected interviewees from the 87 volunteers to represent all three groups, stratifying by BI level and randomly inviting five teachers per group, yielding 15 interviewees, all of whom agreed to participate. We then conducted online semi-structured interviews within one week to support the quantitative findings through triangulation.

**Figure 3:**
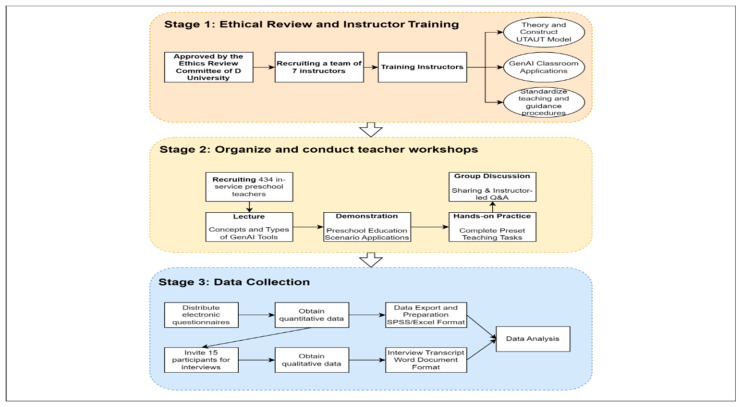
Research Procedure.

The overall research procedure is illustrated in Figure 3.

### 3.2. Participants

We recruited 434 in-service preschool teachers through local education authorities and partner kindergartens using purposive sampling. All participants voluntarily attended professional workshops on GenAI. We distributed and collected questionnaires via Wenjuanxing (www.wjx.cn), a widely used online survey platform in China. After data cleaning (i.e., removing responses completed in less than one minute or with obvious response patterns), 399 valid responses were retained, with a valid response rate of 91.94%.

Demographic characteristics are presented in Table 1. The sample was predominantly female (98.5%), consistent with the early childhood education workforce in China. Teachers aged 26–35 accounted for the largest group (48.12%). Most held a bachelor’s degree (71.18%), and 69.17% had received previous AI or digital technology training.

### 3.3. Instruments

This study employs a mixed-methods research strategy, integrating both quantitative and qualitative research tools to comprehensively and deeply explore the factors influencing preschool teachers’ behavioral intentions toward using GenAI tools.

#### 3.3.1. Questionnaire Design

We adapted the measurement instruments primarily from the UTAUT, drawing on published scales with established reliability and validity. To ensure conceptual accuracy and measurement equivalence of the English-origin scales within the Chinese cultural context, a rigorous “translation–back translation” procedure was implemented. First, a doctoral researcher in English studies conducted the initial translation into Chinese. A second doctoral researcher in applied linguistics, who had no prior exposure to the original scales, independently back-translated the items into English. The research team then compared the original, translated, and back-translated versions, engaging in multiple rounds of discussion and refinement to produce a semantically accurate and naturally phrased Chinese draft.

The final questionnaire consisted of two sections. The first section collected demographic information (gender, age, educational background, teaching experience, and prior training). The second section measured seven constructs using a five-point Likert scale (1 = strongly disagree, 5 = strongly agree). Table 2 summarises each construct, the number of items, and the source from which it was adapted. Full item wording is provided in Appendix A.

#### 3.3.2. Semi-Structured Interview Guide

To address the limitations of quantitative research in capturing the underlying motivations behind teachers’ technology adoption, this study designed a semi-structured interview guide based on the UTAUT theoretical framework as a supplementary qualitative tool. The guide was intended to probe more deeply into how each construct measured in the questionnaire manifests in preschool teachers’ actual work contexts. All interview questions were open-ended, with the full list provided in Appendix B. The interviews were conducted via online video conferencing, each lasting approximately 25–35 min. With participants’ consent, all interviews were audio-recorded and subsequently transcribed and verified to ensure accuracy.

### 3.4. Data Collection and Analysis

Quantitative data were collected primarily through Wenjuanxing. After the completion of the workshops, a survey link was distributed to all 434 participating teachers. The purpose of the study, the voluntary nature of participation, and confidentiality principles were clearly stated on the first page of the questionnaire. A compulsory-response setting was enabled, requiring participants to complete all mandatory items before submission, which effectively minimized missing data and ensured completeness. Over a four-week period, 434 responses were collected. After screening for insufficient response time (less than one minute) and patterned responses, 399 valid questionnaires were retained, thus yielding a valid response rate of 92.79%.

We analysed the quantitative data using SPSS 26.0 and AMOS 26.0. Following the two-step approach recommended by Anderson and Gerbing (1988), we first assessed the measurement model through confirmatory factor analysis (CFA) to evaluate reliability, convergent validity, and discriminant validity. We examined factor loadings, composite reliability (CR), average variance extracted (AVE), and model fit indices including CFI, TLI, RMSEA, and SRMR. After establishing the measurement model’s adequacy, we tested the structural model using path analysis to examine the hypothesized relationships among constructs.

For qualitative analysis, we analyzed the 15 interview transcripts using thematic analysis, following Braun and Clarke’s (2006) six-phase framework.

First, we (first and second authors) familiarized ourselves with the data by reading all transcripts thoroughly. We then independently coded the first five transcripts inductively to generate initial codes, resolved discrepancies through discussion, and developed a preliminary coding framework. Next, we used this framework to code the remaining 10 transcripts independently, then collaboratively grouped codes into potential themes by identifying patterns and relationships. We reviewed and refined these themes against the raw data, modifying or merging them as needed. To assess coding reliability, we randomly selected three transcripts and calculated inter-coder agreement, which averaged 89.3% across all codes—indicating good reliability. We resolved all remaining discrepancies through discussion, and the final themes reflect consensus between both coders. We then defined and named each theme, selected representative quotes, and actively sought disconfirming evidence to ensure balanced findings. Finally, we organized the final themes within the UTAUT framework to integrate with quantitative results. To enhance trustworthiness, five participants verified that the preliminary findings accurately reflected their experiences.

We acknowledge that the single-source, cross-sectional survey design may inflate associations and limit causal claims. The quantitative results are therefore interpreted with caution, and the qualitative findings provide complementary explanatory depth.

## 4. Results

This study employed structural equation modeling to explore the proposed hypotheses, following the two-step approach recommended by Anderson and Gerbing (1988). First, the measurement model was evaluated through reliability and validity assessments as well as model fit indices to assess the robustness and accuracy of the construct measurements. Subsequently, the structural model was analyzed to examine the proposed relationships among variables and to estimate the corresponding path coefficients.

### 4.1. Measurement Model

#### 4.1.1. Multicollinearity Diagnostics

In behavioral science research, multicollinearity is a critical concern because it can lead to unstable parameter estimates and undermine the reliability of statistical inferences. Therefore, prior to conducting the structural equation modeling analyses, this study first assessed multicollinearity among the independent variables to verify that no excessive intercorrelations would compromise the stability of regression coefficient estimation (see Table 3). We evaluated multicollinearity using the Variance Inflation Factor (VIF) and tolerance values. A VIF greater than 10 is commonly considered indicative of severe multicollinearity (Kyriazos & Poga, 2023), while a tolerance value below 0.1 signals a significant multicollinearity problem (Kutner, 2005). The results showed that all constructs had VIF values well below the conventional threshold of 10, and tolerance values were all above 0.1. These findings indicate that the data used in this study do not suffer from severe multicollinearity issues, supporting the appropriateness of proceeding with subsequent analyses.

#### 4.1.2. Descriptive Statistics

Prior to testing the measurement model, we examined the distributional properties of the data. As shown in Table 4, the mean scores for all constructs ranged from 3.57 to 4.44 on a five-point scale, with standard deviations between 0.72 and 1.04, indicating generally positive perceptions of GenAI among participants. To assess univariate normality, we calculated skewness and kurtosis for each construct. Skewness values ranged from −1.067 to −0.026, with absolute values mostly below 1, suggesting a mild left skew where scores clustered toward the higher end. Kurtosis values ranged from −0.835 to 0.505, with all absolute values below 1, indicating that the distributions do not deviate substantially from normality in terms of peakedness. Visual inspection of Q-Q plots and P-P plots further confirmed that the data distributions approximate normality. Given the sample size of 399 and the robustness of structural equation modeling to minor non-normality, the data are suitable for the planned parametric analyses.

#### 4.1.3. Reliability and Validity Analysis

This study assessed the internal consistency reliability of the six constructs included in the analysis. Internal consistency was evaluated using Cronbach’s alpha, which reflects the degree of interrelatedness among the items and indicates whether they measure the same underlying latent construct (N. Ahmad et al., 2024). Values above 0.80 are generally considered indicative of good reliability, while values exceeding 0.90 denote excellent reliability (George & Mallery, 2003). As shown in Table 4, all constructs had Cronbach’s alpha coefficients above the 0.80 threshold, suggesting strong internal consistency for each scale.

To assess the measurement model’s convergent validity and discriminant validity, we conducted confirmatory factor analysis (CFA). CFA results suggest that all factor loadings were statistically significant (*p* < 0.001), with standardized values substantially exceeding the generally accepted threshold of 0.5. As shown in Table 5, the composite reliability (CR) for each construct ranged from 0.860 to 0.962, exceeding the recommended threshold of 0.7. The average variance extracted (AVE) exceeded 0.5 for all constructs and remained below their respective composite reliability values. Collectively, these results suggest that the measurement model possesses strong convergent validity (Fornell & Larcker, 1981).

Regarding discriminant validity, we compared the square root of each construct’s AVE with the absolute values of the inter-construct correlations. The results suggested that, except for effort expectancy (EE)—whose AVE square root was slightly lower than its correlation with performance expectancy (PE)—all constructs had AVE square roots greater than their correlations with other constructs. Considering that the composite reliability of EE (CR = 0.929) and its maximal reliability (MaxR(H) = 0.936) were both well above the 0.70 threshold, suggesting strong internal consistency, and given the well-established theoretical linkage between EE and PE in the UTAUT framework, this deviation is considered acceptable. In addition, we assessed discriminant validity using the heterotrait–monotrait ratio (HTMT). As shown in Table 6, all HTMT values were below the recommended cutoff of 0.90 (Henseler et al., 2015), further suggesting adequate discriminant validity across all constructs.

Table 7 compares the model fit indices with the benchmark criteria recommended by Hair et al. (2010), and the results suggest that all fit indices fall within the suggested thresholds. These findings suggest that the model shows a reasonable level of fit with the observed data, further suggesting the validity of the measurement scales and providing preliminary support for the subsequent path analysis.

### 4.2. Structural Model Analysis

Using path analysis, this study explored the associations between the constructs. Given the high correlations among some predictors (e.g., PE and EE) and potential common method variance, these results should be viewed as exploratory. As shown in Table 8, hypotheses H1, H3, H5, and H6 received provisional support, whereas H2 and H4 did not. The model explains 71.3% of the variance in behavioral intention (R^2^ = 0.713), but this high explanatory power may be partially inflated by common method bias. We therefore place greater interpretive weight on the convergence between quantitative patterns and qualitative themes.

### 4.3. Preschool Teachers’ Interview Results

To explore the factors shaping preschool teachers’ behavioral intention to use GenAI, this study conducted a thematic analysis of 15 interview transcripts. Following Braun and Clarke’s (2006) six-phase framework, we carried out the analysis within the UTAUT framework. The major themes, subthemes, and representative excerpts are summarized in Table 9.

Drawing on these findings, the qualitative results demonstrate that preschool teachers’ intention to adopt GenAI is primarily driven by three forces: performance expectancy (perceived usefulness of the tools), social influence (advocacy from peers and organizational stakeholders), and tech-savviness (confidence in one’s own technological competence). At the same time, perceived risks (concerns about content accuracy and data safety) constitute the most significant barrier to adoption.

The qualitative data are not subject to the same discriminant validity or common methodological concerns that affect the survey, because they capture teachers’ own words and reasoning independently of the quantitative scales. Thus, the consistent direction of qualitative accounts provides a more trustworthy source of insight into teachers’ decision-making processes. More importantly, they explain why effort expectancy and facilitating conditions did not show significant associations: teachers generally believe that the high value returns offered by GenAI justify bearing certain learning costs. Meanwhile, the widespread availability of smartphones and internet access has made basic technological conditions a “default configuration,” thereby rendering these factors non-core considerations in their decision-making. In summary, whether teachers adopt GenAI hinges on whether the driving forces are sufficiently compelling to overcome internal risk concerns and convince them that the learning costs incurred are worthwhile.

## 5. Discussion

This study explores the factors associated with preschool teachers’ behavioral intentions to use GenAI tools for teaching and research, based on an extended UTAUT model. It suggests possible mechanisms underlying technology adoption within this group, providing preliminary insights into their cognitive and behavioral processes.

### 5.1. Interpretation of Supporting Hypotheses (H1, H3, H5, H6)

#### 5.1.1. Impact of Performance Expectancy

Performance expectancy suggests a significant positive association with preschool teachers’ behavioral intention to use GenAI (β = 0.43, *p* < 0.001), a finding consistent with previous research (Li & Zhang, 2024; An et al., 2022; Ifedayo et al., 2020). In educational contexts, teachers’ adoption of new technologies is typically related to motives such as enhancing instructional efficiency, improving content quality, and supporting individualized learning (Chatterjee & Bhattacharjee, 2020). When teachers are confident that a tool can substantively improve their work performance, their willingness to adopt it tends to be higher.

The interview data support this quantitative pattern. One teacher remarked: “Previously, preparing materials for a single teaching activity consumed an entire weekend. Now, by inputting keywords into AI, I can generate multiple visual resources within minutes.” Another teacher highlighted the creative dimension: “It offers many activity ideas I hadn’t thought of. It also helps me organize my logical framework when writing papers.” These accounts suggest that when teachers clearly perceive GenAI’s effectiveness in producing customized materials, supporting instructional design, and stimulating creativity, their intention to adopt the technology tends to be stronger. For educational practitioners, this finding suggests the importance of demonstrating concrete efficiency gains and creative applications in professional development programmes, helping teachers see tangible value in their daily work.

#### 5.1.2. Impact of Social Influence

Social influence suggests a positive association with preschool teachers’ behavioral intentions toward using GenAI tools (β = 0.37, *p* < 0.001), consistent with findings in the field of educational technology adoption (Li & Zhang, 2024; Xu et al., 2025; Du & Lv, 2024; Yakubu et al., 2025).

The interviews further suggested that peer modeling and administrative support appeared as important external factors, aligning closely with the quantitative result. As one teacher noted: “I was actually hesitant about these AI tools at first, but when I saw several teachers using them and the principal encouraging us to give them a try, I started experimenting with using AI to design instructional activities. I soon realized that it was indeed very helpful.” Another described peer pressure: “All the teachers around me are using it, and I feel like I’ll be left behind if I don’t.” These accounts suggest that, within school settings, leadership advocacy and a collegial climate of use are associated with stronger behavioral intention to adopt GenAI. Kindergarten leaders can actively foster a supportive climate by encouraging GenAI use and leveraging early adopters as peer models. Establishing professional learning communities where teachers share experiences may further support this influence.

#### 5.1.3. Impact of Perceived Risks

Perceived risk suggests a significant negative association with preschool teachers’ behavioral intentions toward using GenAI (β = −0.118, *p* = 0.02), a finding consistent with technology adoption patterns in fields such as eHealthcare and FinTech (Arfi et al., 2021; Amnas et al., 2023). The distinctive characteristics of early childhood education further make this association particularly salient, as teachers hold higher expectancy regarding the accuracy of instructional content, the safety of learning materials, and the protection of children’s data privacy (Wang et al., 2024).

The interviews reflect these concerns, providing depth to the quantitative findings. One interviewee articulated: “I do worry about this. For example, if I ask AI to generate a science story about animals, what if it includes inaccurate information or details that might frighten the children? And now we are required to be very cautious about protecting children’s privacy. Uploading their information to a third-party platform never feels completely secure.” Another teacher added: “What I dread most is that the knowledge it generates might be incorrect, forcing me to double-check it myself.” These concerns suggest that perceived risks related to content accuracy and data security are strongly associated with lower GenAI adoption intentions. Addressing these concerns requires institutions to provide clear guidelines on responsible GenAI use, transparent data protection protocols, and training on evaluating AI-generated content—measures that can help teachers feel more confident about safety and accuracy of GenAI tools.

#### 5.1.4. Impact of Tech-Savviness

Tech-savviness suggests a significant positive association with preschool teachers’ behavioral intention to use GenAI (β = 0.495, *p* < 0.001), which appears to be the strongest predictor in the model. When teachers possess stronger operational skills and greater confidence in their ability to navigate the technology, they report stronger intentions to integrate GenAI proactively into their teaching practices.

This pattern was also observed in the interview data. A teacher with high tech-savviness explained: “Since I’m always tinkering with new software, I pick things up quickly. That feeling of ‘I can handle this’ makes me eager to use it.” Conversely, a less confident teacher described frustration: “Sometimes I don’t know how to ask questions to get the results I want. After a few unsuccessful attempts, I start to feel discouraged.” These contrasting accounts are consistent with the quantitative finding that higher digital competence is associated with stronger adoption intentions.

Existing research provides theoretical support for this observed relationship. Wang et al. (2024) demonstrated a significant association between teachers’ technology self-efficacy and their behavioral intention to design GenAI-assisted instruction. In addition, more technologically proficient users are better able to leverage the capabilities of digital tools and obtain more positive user experiences (Demir & Demir, 2023; M. Zhang et al., 2022). Thus, higher levels of tech-savviness may help reduce uncertainty and operational burden during use, contributing to teachers’ sense of control and perceived effectiveness. This suggests that building teachers’ digital competence through hands-on workshops, ongoing technical support, and peer learning opportunities should be a priority for institutions seeking to promote GenAI adoption.

### 5.2. Interpretation of Unsupported Hypotheses (H2, H4)

Sampling bias should be considered when interpreting the hypotheses. As noted in the methodology, participants were recruited through a GenAI-focused professional workshop, likely selecting those with greater tech interest or institutional support, as shown by high mean scores: performance expectancy (4.44/5), effort expectancy (4.24/5), and social influence (4.32/5). This ceiling effect and restricted variance may have reduced statistical power, contributing to the non-significant findings for effort expectancy and facilitating conditions. The non-significance of H2 and H4 may thus reflect a range restriction artefact, not their actual irrelevance in the wider population. Caution is needed when generalizing to teachers with less tech exposure or fewer resources, where these factors could show stronger associations.

#### 5.2.1. Impact of Effort Expectancy

This study suggests that effort expectancy is not significantly associated with preschool teachers’ behavioral intention to use GenAI. Similar to our findings, Wu et al. (2025) also observed that effort expectancy had no significant association with behavioral intentions regarding GenAI acceptance among physical education students.

The interview data provide a direct explanation for this result, suggesting why the quantitative path was non-significant. As one teacher remarked: “Actually, whether it’s easy to operate isn’t the main issue. If it can help me, spending some time learning it is no big deal.” Another teacher noted: “At first, you have to learn how to ‘give commands’, but it’s just like searching in a browser—you’ll figure it out after a few tries. It’s not that complicated.” This suggests that when the practical value of the technology is sufficiently salient, teachers are willing to tolerate a certain learning cost, thereby diminishing the relative importance of “ease of use” in their decision-making.

Although numerous studies report a positive association between effort expectancy and behavioral intentions (J. Liu et al., 2024; Ifedayo et al., 2020), the non-significance observed in this study may be further explained by China’s collectivist cultural context. In collectivist societies, teachers often approach new challenges through collaborative problem-solving and peer support (Tiffany & Rosman, 2024). Within Chinese kindergartens, it is common for teachers to share tips, troubleshoot together, and learn new technologies as a community. This collective approach to technology adoption can effectively diffuse the perceived difficulty of learning GenAI tools, as individual teachers do not bear the burden of mastering the technology alone. When difficulties arise, they can readily seek assistance from colleagues, reducing the personal cost associated with effort expectancy and diminishing its role as a key factor associated with adoption intentions.

#### 5.2.2. Impact of Facilitating Conditions

This study suggests that facilitating conditions are not significantly associated with preschool teachers’ behavioral intention to use GenAI, a result that diverges from most existing research (Wu et al., 2025). The interview data offer a compelling explanation for this discrepancy, again providing qualitative depth to the statistical outcome. As one teacher noted: “Using these AI tools is very convenient now. I can access them directly on my phone, and the kindergarten’s network is more than sufficient. Honestly, as long as I want to use them, we basically have everything we need.” Another teacher added: “To be honest, whether there is training or not doesn’t affect my use that much. What matters is whether I personally want to use it.”

These findings suggest that when basic technical infrastructure is widely available, teachers’ adoption intentions are more strongly related to personal motivation and perceived tool value than to external support. Modern GenAI tools such as ChatGPT, DeepSeek, and Doubao feature user-friendly, conversational interfaces that lower technical barriers. Intuitive and accessible technology may reduce the relevance of external facilitating conditions like technical support or institutional resources. In well-supported environments, such facilitating conditions appear to shift from critical determinants to basic provisions, reducing their observed associations with behavioral intention. In this study, 69.17% of teachers received relevant training, suggesting that facilitating conditions were generally sufficient and no longer showed a strong association with behavioral intention.

While effort expectancy and facilitating conditions were not significant in this sample, practitioners should not dismiss them entirely. In less-resourced settings or among teachers with limited digital experience, these factors may still be important. This contrast is evident when comparing our findings with Şakır (2025), who found that both effort expectancy and facilitating conditions significantly predicted Turkish preschool teachers’ technology adoption, suggesting that the influence of these factors is context-dependent on infrastructural maturity and cultural norms. Therefore, ongoing investment in infrastructure and user support remains important, even if it does not show a direct association with intention in already-advantaged preschool teacher populations.

### 5.3. Overall Interpretive Caution and the Value of Qualitative Insights

Taken together, the quantitative findings should be viewed as exploratory rather than confirmatory. High inter-construct correlations (notably between PE and EE) raise discriminant validity concerns, and the single-source cross-sectional design may inflate associations via common method variance. Thus, path coefficients may represent upper-bound estimates, and non-significant results for EE and FC could reflect statistical artefacts such as multicollinearity or range restriction.

By contrast, the qualitative data are less affected by these statistical limitations. They offer consistent contextual evidence that performance expectancy, social influence, and tech-savviness promote GenAI adoption, whereas perceived risks constrain it; effort expectancy and facilitating conditions play a weaker role when performance benefits are clear and basic infrastructure is available. The most reliable contribution of this study therefore lies in the convergent evidence from mixed-methods analysis and the qualitative insights explaining the salience of different adoption factors.

## 6. Conclusions

This study presents an exploratory, mixed-methods investigation of preschool teachers’ intentions to use GenAI. Despite limitations related to discriminant validity and common method bias, the quantitative analyses suggest positive associations of intention with performance expectancy, social influence, and tech-savviness, as well as a negative association with perceived risks. Effort expectancy and facilitating conditions showed no significant associations, patterns that are further explained by the qualitative data.

Due to the aforementioned methodological limitations, we do not claim that these findings are conclusive. Instead, we propose them as hypotheses for future confirmatory research. The qualitative interviews independently support the direction of these patterns and offer practical insights: teachers prioritize performance benefits, are influenced by social encouragement, rely on digital competence, and express concerns about potential risks. By contrast, effort expectancy and facilitating conditions appear less critical in well-resourced, collectivist contexts.

Practitioners may still consider the qualitative lessons as actionable, but they should await stronger quantitative evidence. Theoretically, this study contributes by extending UTAUT to preschool education and highlighting contextual boundary conditions (infrastructure maturity, collectivist culture) that may moderate the relevance of effort expectancy and facilitating conditions. Future research should adopt multi-source, longitudinal designs, employ measures with improved discriminant validity, and recruit larger, more representative samples to formally test the hypotheses proposed in this study.

## 7. Research Limitations and Future Research

Despite rigorous procedures, this study has several limitations. First, participants were recruited via GenAI-related workshops, which may introduce self-selection bias. The generally high mean scores (e.g., performance expectancy = 4.44/5) suggest potential ceiling effects, which may have reduced variance and contributed to the non-significant findings for effort expectancy and facilitating conditions. Thus, the results may not fully represent the broader preschool teacher population, especially those in resource-limited settings. Second, the cross-sectional design only reflects intentions at one time point, which limits understanding of long-term changes in attitudes and behaviors. All quantitative data were self-reported, raising concerns about common method variance. Harman’s single-factor test showed that the first factor explained 51.99% of the variance. Although this test is a relatively weak diagnostic, the fact that a single factor accounts for more than 40% of the variance should be treated as a warning sign. Therefore, common method bias may have inflated some of the observed correlations, and the structural path coefficients should be interpreted as upper-bound estimates. Third, effort expectancy and performance expectancy were highly correlated (r = 0.884), raising discriminant validity concerns. Although HTMT analysis supported their distinctiveness, their close relationship may have affected the non-significant result of H2, which could partly reflect statistical overlap rather than theoretical irrelevance. This degree of overlap suggests that the survey instrument may not have adequately distinguished these two theoretical constructs. The non-significant finding for H2 therefore does not indicate that effort expectancy is unimportant, but may instead reflect a measurement artifact. Future research should use more refined scales or experimental designs to better separate PE and EE. Although we cannot statistically rule out common method bias, the consistency of the qualitative findings with the quantitative patterns provides a form of triangulation that reduces the likelihood that the observed relationships are purely method-induced.

Future research should address these limitations through longitudinal designs tracking teachers’ attitudes over time, broader sampling strategies including diverse regions and under-resourced settings, and cross-cultural comparisons to examine whether patterns observed in China’s collectivist context hold in individualist societies. Procedural remedies such as temporal separation of measurements or multi-source data collection would help minimize CMV, and more refined measures could better distinguish theoretically related constructs like effort expectancy and performance expectancy.

## Figures and Tables

**Table 1:** Demographic statistics (*n* = 399).

Demographic	Characteristics	Frequency	Percentage (%)
Gender	Male	6	1.50
	Female	393	98.50
Age	25 years old or below	71	17.79
	26–30 years old	100	25.06
	31–35 years old	92	23.06
	36–40 years old	65	16.29
	41 years old or above	71	17.79
Highest Educational Qualification	High school diploma or below	12	3.01
	Associate degree	88	22.06
	Bachelor’s degree	284	71.18
	Master’s degree or above	15	3.76
Teaching Experience	Less than 2 years	48	12.03
	2–3 years	50	12.53
	4–5 years	42	10.53
	6–10 years	107	26.82
	More than 10 years	152	38.10
Have received training related to AI or digital technology	Yes	276	69.17
	No	123	30.83

**Table 2:** Summary of Constructs, Items, and Sources.

Construct	Number of Items	Adapted Source
Performance Expectancy (PE)	4	Venkatesh et al. (2003); Yakubu et al. (2025)
Effort Expectancy (EE)	4	Venkatesh et al. (2003); Yakubu et al. (2025)
Social Influence (SI)	4	Venkatesh et al. (2003); Yakubu et al. (2025)
Facilitating Conditions (FC)	4	Venkatesh et al. (2003); Yakubu et al. (2025)
Perceived Risk (PR)	3	Yakubu et al. (2025)
Tech-Savviness (TS)	3	Guha et al. (2023)
Behavioral Intention (BI)	4	Venkatesh et al. (2003); Yakubu et al. (2025)

**Table 3:** Multicollinearity results.

Construct	Tolerance	VIF
PE	0.212	4.708
EE	0.204	4.905
SI	0.246	4.057
FC	0.289	3.458
PR	0.601	1.663
TS	0.38	2.633

**Note(s):** Dependent variable: Behavioral Intention (BI).

**Table 4:** Reliability of constructs.

Construct	Mean	SD	Cronbach’s Alpha	Number of Items
PE	4.442	0.720	0.958	4
EE	4.242	0.774	0.927	4
SI	4.323	0.758	0.962	4
FC	4.028	0.863	0.946	4
PR	3.595	1.023	0.855	3
TS	3.571	1.036	0.908	3
BI	4.202	0.799	0.968	4
Total scale	4.094	0.682	0.967	26

**Table 5:** Reliabilities and correlations.

	CR	AVE	MSV	MaxR(H)	PE	EE	SI	FC	PR	TS
PE	0.959	0.853	0.781	0.959	0.924					
EE	0.929	0.766	0.781	0.936	0.884 ***	0.875				
SI	0.962	0.865	0.748	0.963	0.865 ***	0.830 ***	0.93			
FC	0.947	0.816	0.668	0.948	0.682 ***	0.818 ***	0.766 ***	0.903		
PR	0.86	0.674	0.469	0.886	0.300 ***	0.345 ***	0.289 ***	0.413 ***	0.821	
TS	0.912	0.776	0.523	0.915	0.492 ***	0.640 ***	0.524 ***	0.723 ***	0.685 ***	0.881

**Note(s):** CR = Composite Reliability; CR = Composite Reliability; MSV = Maximum Shared Variance; MaxR(H) = Maximum Reliability (Henseler’s); Diagonal lines rendered in italic face are the square roots of AVEs. *** means significant correlation at *p* < 0.001.

**Table 6:** HTMT Analysis.

	PE	EE	SI	FC	PR
PE	1				
EE	0.888	1			
SI	0.864	0.829	1		
FC	0.688	0.824	0.769	1	
PR	0.354	0.397	0.346	0.455	1
TS	0.492	0.646	0.527	0.729	0.702

**Table 7:** Model Fit Summary Relating to the Research Model.

Fit Index	Recommended Value	Value in the Model
Chi-Square	N/A	958.145
Degree of Freedom (df)	N/A	278
Comparative Fit Index (CFI)	≥0.90	0.947
Tucker–Lewis Index (TLI)	≥0.90	0.938
Normed Fit Index (NFI)	≥0.90	0.928
Root Mean Square Error of Approximation (RMSEA)	<0.08	0.078
Incremental Fit Index (IFI)	≥0.90	0.948
Standardized Root Mean Square Residual (SRMR)	<0.08	0.054

**Table 8:** Structural Model.

Hypothesis	Path	Estimate	*p*-Value	R^2^	Outcome
H1	PE→BI	0.43	***	0.713	Support
H2	EE→BI	−0.187	0.100		Unsupport
H3	SI→BI	0.37	***		Support
H4	FC→BI	−0.06	0.432		Unsupport
H5	PR→BI	−0.118	0.020		Support
H6	TS→BI	0.495	***		Support

**Note(s):** *** means significant correlation at *p* < 0.001.

**Table 9:** Thematic Analysis of Factors Influencing Preschool Teachers’ Intentions to Use GenAI.

Theme	Subtheme	Representative Interview Quote
Performance Expectancy	1.1 Enhanced efficiency in lesson preparation	It’s so much more efficient for lesson planning—it generates a complete draft in just one minute, and I only need to make minor adjustments.
	1.2 Stimulating instructional creativity and supporting research work	It offers many activity ideas I hadn’t thought of. It also helps me organize my logical framework when writing papers.
Effort Expectancy	2.1 Easy to operate and quick to learn	At first, you have to learn how to ‘give commands’, but it’s just like searching in a browser—you’ll figure it out after a few tries. It’s not that complicated.
	2.2 Ease of use does not determine willingness to adopt	Actually, whether it’s easy to operate isn’t the main issue. If it can help me, spending some time learning it is no big deal.
Social Influence	3.1 Peer modeling and conformity pressure	All the teachers around me are using it, and I feel like I’ll be left behind if I don’t.
	3.2 Explicit encouragement from administrators	The principal encouraged us to try it at the meeting, which made me feel that using this approach was supported and affirmed.
Facilitating Conditions	4.1 Basic technological conditions are already in place	Everyone has a smartphone and internet access now. The basic conditions for using it are essentially met.
	4.2 Systematic training remains insufficient	The kindergarten has offered some training, but it’s not systematic. Most of the time, I explore it on my own, and sometimes I don’t know whom to ask when problems arise.
	4.3 Weak linkage between support conditions and intention to use	To be honest, whether there is training or not doesn’t affect my use that much. What matters is whether I personally want to use it.
Perceived Risk	5.1 Concerns about data security	I dare not enter any child’s personal information, not even their name—I just can’t shake the feeling that storing it on someone else’s server isn’t safe.
	5.2 Risks associated with content accuracy and professional appropriateness	What I dread most is that the knowledge it generates might be incorrect, forcing me to double-check it myself.
Tech-savviness	6.1 Proficiency boosts confidence in use	Since I’m always tinkering with new software, I pick things up quickly. That feeling of ‘I can handle this’ makes me eager to use it.
	6.2 Lack of familiarity leads to frustration and reduced use	Sometimes I don’t know how to ask questions to get the results I want. After a few unsuccessful attempts, I start to feel discouraged.

## Data Availability

The data presented in this study are available on request from the corresponding author due to ethical restrictions.

## Appendix A. Questionnaire

Dear teachers,

We are researchers working in the field of early childhood education. We are conducting a study on preschool teachers’ use of artificial intelligence (AI) tools, with a particular focus on generative AI (GenAI). In this survey, “Generative Artificial Intelligence” will be referred to as GenAI. It denotes artificial intelligence systems capable of generating content like text, images, and code, such as DeepSeek, Doubao, Tencent Yuanbao, ChatGPT, etc. Your responses are essential for us to better understand how AI tools are being perceived and used in preschool education. We assure you that all the information you provide will be kept strictly confidential and will be used for research purposes only.

Part 1: Background Information

1. Gender

□ Male

□ Female

2. Age

□ 25 years old or below

□ 26–30 years old

□ 31–35 years old

□ 36–40 years old

□ 41 years old or above

3. Highest Educational Qualification

□ High school diploma or below

□ Associate degree

□ Bachelor’s degree

□ Master’s degree or above

4. Teaching Experience

□ Less than 2 year

□ 2–3 years

□ 4–5 years

□ 6–10 years

□ More than 10 years

5. Have you ever received training related to Artificial Intelligence or digital technology?

□ Yes

□ No

Part 2: Teachers’ Perceptions on the Use of Generative AI Tools for Teaching and Research

Please blacken the following ratings below, indicating 1 (absolutely disagree), 2 (disagree), 3 (neutral), 4 (agree) and 5 (absolutely agree)

**Factors**	**Items**	**Scales**				
Performance Expectancy (PE)	PE1: I find GenAI tools useful in my teaching and research.	1	2	3	4	5
	PE2: Using Al technologies enables me to accomplish tasks more quickly.	1	2	3	4	5
	PE3: Using Al technologies increases my productivity.	1	2	3	4	5
	PE4: If I use generative AI technologies, I will increase my chances of achieving better teaching and research performance.	1	2	3	4	5
Effort Expectancy (EE)	EE1: My interaction with GenAI technologies is clear and understandable.	1	2	3	4	5
	EE2: It is easy for me to become skillful at using GenAI technologies.	1	2	3	4	5
	EE3: I find GenAI technologies easy to use.	1	2	3	4	5
	EE4: Learning to operate GenAI tools is easy for me.	1	2	3	4	5
Social Influence (SI)	SI1: My colleagues encourage me to use GenAI technologies for teaching and research.	1	2	3	4	5
	SI2: My peers in the preschool education field encourage me to use GenAI technologies for teaching and research.	1	2	3	4	5
	SI3: The kindergarten (management/administration) encourage me to use GenAI technologies for teaching and research.	1	2	3	4	5
	SI4: Generally speaking, People who are important to me think that I should use GenAI technologies for teaching and research.	1	2	3	4	5
Facilitating Conditions (FC)	FC1: I have all the necessary resources to use GenAI tools for teaching and research.	1	2	3	4	5
	FC2: I have the knowledge necessary to use GenAI tools in teaching and research.	1	2	3	4	5
	FC3: My kindergarten or colleagues are available to assist me with using GenAI tools in teaching and research.	1	2	3	4	5
	FC4: My workplace has all the necessary resources to use GenAI technologies for teaching and research.	1	2	3	4	5
Perceived Risk (PR)	PR1: I believe the content generated by GenAI tools is not always reliable.	1	2	3	4	5
	PR2: The use of GenAI for teaching and research purpose is confusing.	1	2	3	4	5
	PR3: Use of GenAI technology for teaching and research purpose is risky.	1	2	3	4	5
Tech-savviness (TS)	TS1: I am constantly being sought after by people for advice on new GenAI technology.	1	2	3	4	5
	TS2: I am typically one of the first to use new GenAI technology when it appears.	1	2	3	4	5
	TS3: I am able to use multiple GenAI technologies with ease.	1	2	3	4	5
Behavioral Intentions (BI)	BI1: I intend to use GenAI tools in my teaching and research in the near future.	1	2	3	4	5
	BI2: I predict I will use GenAI tools in my teaching and research in the near future.	1	2	3	4	5
	BI3: I plan to use GenAI tools frequently in my teaching and research.	1	2	3	4	5
	BI4: When given the chance, I will always try to use GenAI tools in my teaching and research.	1	2	3	4	5

## Appendix B. Semi-Structured Interview Guide

1. In what ways do you believe GenAI tools can enhance your teaching or research efficiency? Specifically, which tasks or activities do you feel they could assist with more effectively?

2. How easy do you find GenAI tools to operate? If you find them easy to use, what makes them feel effortless? If you encounter difficulties, where do you see the main challenges?

3. Our survey data indicates that “ease of use” is not a key factor influencing teachers’ willingness to adopt the tool. In your view, why is its “usability” relatively less important?

4. Do you feel that expectancy from colleagues or your institution have influenced your use of GenAI tools? To what extent has this social pressure affected your attitudes and behaviors?

5. Do you feel the technical support provided by your school or institution is sufficient? If not, what aspects of support are most lacking, causing you to hesitate in using the tools?

6. Our research reveals that even when some of the aforementioned support conditions are provided, it does not necessarily lead to teachers’ willingness to use the tool. In your view, why is this the case?

7. Do you have concerns about data security or content accuracy when using GenAI tools? To what extent do these concerns influence your decision to use them?

8. Do you consider yourself sufficiently tech-savvy to use GenAI tools? If you encounter difficulties, what specific technical issues hinder your usage?
